# Peptides derived from the HIV-1 integrase promote HIV-1 infection and multi-integration of viral cDNA in LEDGF/p75-knockdown cells

**DOI:** 10.1186/1743-422X-7-177

**Published:** 2010-08-02

**Authors:** Aviad Levin, Zvi Hayouka, Assaf Friedler, Abraham Loyter

**Affiliations:** 1 Department of Biological Chemistry, The Alexander Silberman Institute of Life Sciences; The Hebrew University of Jerusalem, Safra Campus, Givat Ram, Jerusalem 91904, Israel; 2 Institute of Chemistry; The Hebrew University of Jerusalem, Safra Campus, Givat Ram, Jerusalem 91904, Israel

## Abstract

**Background:**

The presence of the cellular Lens Epithelium Derived Growth Factor p75 (LEDGF/p75) protein is essential for integration of the Human immunodeficiency virus type 1 (HIV-1) cDNA and for efficient virus production. In the absence of LEDGF/p75 very little integration and virus production can be detected, as was demonstrated using LEDGF/p75-knokdown cells.

**Results:**

Here we show that the failure to infect LEDGF/p75-knockdown cells has another reason aside from the lack of LEDGF/p75. It is also due to inhibition of the viral integrase (IN) enzymatic activity by an early expressed viral Rev protein. The formation of an inhibitory Rev-IN complex in virus-infected cells can be disrupted by the addition of three IN-derived, cell-permeable peptides, designated INr (IN derived-Rev interacting peptides) and INS (IN derived-integrase stimulatory peptide). The results of the present work confirm previous results showing that HIV-1 fails to infect LEDGF/p75-knockdown cells. However, in the presence of INrs and INS peptides, relatively high levels of viral cDNA integration as well as productive virus infection were obtained following infection by a wild type (WT) HIV-1 of LEDGF/p75-knockdown cells.

**Conclusions:**

It appears that the lack of integration observed in HIV-1 infected LEDGF/p75-knockdown cells is due mainly to the inhibitory effect of Rev following the formation of a Rev-IN complex. Disruption of this inhibitory complex leads to productive infection in those cells.

## Background

Productive infection of susceptible cells by Human immunodeficiency virus type 1 (HIV-1) has been shown to require, in addition to virus-encoded proteins, the presence of the host cellular protein Lens Epithelium Derived Growth Factor p75 (LEDGF/p75) [1-3]. Following nuclear import of a viral integrase (IN)-DNA complex, IN interacts with intranuclear LEDGF/p75 molecules, which pave its way via the recipient cells chromatin allowing efficient integration [1,4-6]. This is mediated by the LEDGF/p75 AT hook and PWWP domains [7-9]. The requirement for LEDGF/p75 was demonstrated by experiments showing a lack of integration, and thus virus production, in LEDGF/p75-knockdown cells [4,6,10,11]. Moreover, expression of the LEDGF/p75 integrase-binding domain (IBD), which mediates the LEDGF/p75 binding to IN, was shown to significantly inhibit integration and virus infection due to its ability to interfere with the IN-LEDGF/p75 interaction [12]. Finally, HIV strains bearing mutated IN proteins which fail to interact with LEDGF/p75 are not infectious [13]. These results demonstrate that the presence of intracellular LEDGF/p75 protein is essential for efficient virus infection. However, integration of HIV-1 cDNA can occur in LEDGF/p75-knockdown cells following infection with HIV-1 mutant lacking the Rev protein (ΔRev virus), as has been shown previously by us [14].

Following integration of the viral cDNA, several viral proteins are expressed, among them Rev [15]. After its nuclear import the Rev protein is involved in nuclear export of unspliced and partially spliced viral RNA molecules [15]. Thus, similar to IN, the presence the Rev protein is essential for completion of the HIV-1 life cycle [15]. In addition to its expression from integrated viral DNA, Rev can be expressed from unintegrated DNA molecules and thus appear at an early stage in virus-infected cells [16-20]. Recently, we have shown that early expressed Rev can interact with IN in virus-infected cells, resulting in inhibition of IN nuclear import [18,21] as well as of its enzymatic activity [17,22,23]. Rev-induced inhibition of the IN enzymatic activity resulted in inhibition of cDNA integration and significant reduction in the degree of virus infection [14,17,24]. Formation of the Rev-IN complex in virus-infected cells can be disrupted by three cell-permeable IN-derived peptides, the INrs (IN derived-Rev interacting peptides) [22] and INS (IN derived-integrase stimulatory peptide) [25]. The INS, in addition to its ability to promote dissociation of the Rev-IN complex, was able to stimulate the enzymatic activity of the IN itself *in vitro*, and consequently the integration of viral cDNA in virus infected cells [25].

In the current work we show that in the presence of the INr and INS peptides, WT HIV-1 can productively infect LEDGF/p75-knockdown cells. Furthermore, a relatively high degree of viral cDNA integration was observed in these cells following their incubation with the INr and INS peptides. These results indicate that the previously reported [4,6,10,11] failure of the HIV-1 to infect LEDGF/p75-knockdown is mainly due to the formation of the inhibitory Rev-IN complex.

## Results

### The INS peptide binds to LEDGF/p75 and partially disrupts the IN-LEDGF/p75 complex

**Figure 1:**
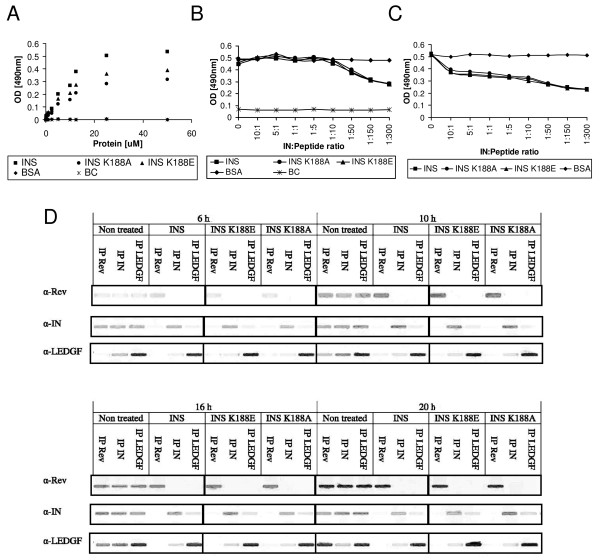
**INS and INS-derived peptides bind LEDGF/p75 and promote partial dissociation of the IN-LEDGF/p75 complex**. **(A) **LEDGF/p75 was incubated in ELISA plates coated with the indicated peptide or with BSA as a negative control, and binding was determined as described in Methods. Wells containing the buffer carbonate (BC) in the absence of peptide were used as a background control. **(B) **The IN protein was first bound to the ELISA plates which were then incubated with LEDGF/p75 to obtain LEDGF/p75-IN complex. The complex was then incubated with either the indicated peptide or BSA at the designated IN:peptide (or BSA) ratios. The amount of bound LEDGF/p75 was then determined. **(C) **Same as (B) but the peptides (or BSA) were added at the same time as LEDGF/p75 to determine competition. **(D) **Formation of Rev-IN, IN-LEDGF/p75 and Rev-LEDGF/p75 complexes and their dissociation by the INS and INS-derived peptides. Co-IP was performed in lysates obtained from virus-infected cells. All other experimental conditions are described in Methods.

The INS peptide was derived from the IN domain that mediates IN binding to Rev [25] as well as IN-IN interactions [26]. This peptide stimulates IN enzymatic activity *in vitro *and integration of the viral genome in HIV-1-infected cells [25]. Based on structural studies, it appears that binding of the IN to the LEDGF/p75 protein is also mediated by the same domain [2]. It was therefore of interest to determine whether the INS peptide, in addition to its binding to IN and Rev, is also able to interact with the LEDGF/p75 protein. ELISA binding studies revealed specific binding of INS to LEDGF/p75 (Fig. 1A and Table 1). The same was observed with two modified INS peptides (INS K188E and K188A [25]). The results in Fig. 1B and 1C show that the INS and its two derived peptides caused *in vitro *only partial inhibition of the IN-LEDGF/p75 interaction. Being cell permeable [25], these peptides were able to cause partial disruption of the IN-LEDGF/p75 complex formed in virus infected cells as was revealed by co-immunoprecipitation (Co-IP) experiments of an extract obtained from HIV-infected cells (Fig. 1D).

**Table 1:** Binding to LEDGF/p75.

			LEDGF
			
Peptide	Sequence	IN residues	K_d _[μM]*
INS	WTAVQMAVFIHNFKRK	W+174-188	2.2 ± 0.7
INS K188A	WTAVQMAVFIHNFKRA	W+174-187+A	3.0 ± 1.5
INS K188E	WTAVQMAVFIHNFKRE	W+174-187+E	4.5 ± 2.0

* Apparent *K_d _*according to ELISA system using LEDGF and BSA alone as control.

### The INS peptide promotes HIV-1 cDNA integration in LEDGF/p75-knockdown cells

**Figure 2:**
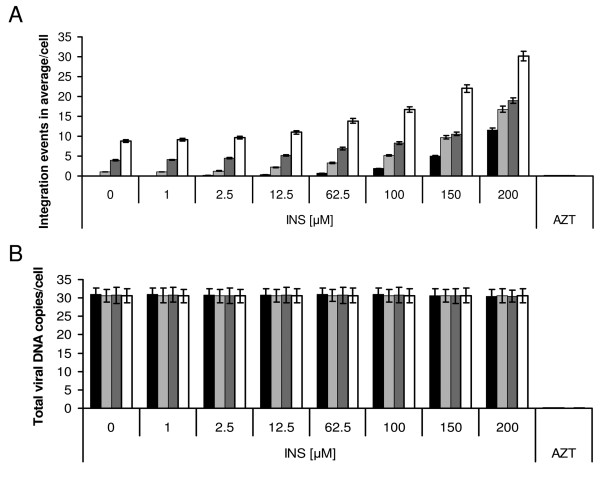
**Effect of INS concentrations on integration and total viral-DNA in infected wt and LEDGF/p75-knockdown HeLa-P4 cells**. HeLa P4 and HeLaP4/shp75Cl15 (LEDGF/p75-knockdown) cells were incubated with the indicated concentration of INS and infected with wt or ΔRev HIV-1 at a MOI of 1.0. The average number of integration events per cell **(A) **and of total viral DNA copies per cell **(B) **was estimated as described in Methods. Black shading and dark grey shading are LEDGF/p75-knockdown cells infected with WT or ΔRev HIV-1, respectively; white grey shading and white shading are HeLa P4 cells infected with WT or ΔRev HIV-1, respectively. AZT was used at 2 μM concentration.

The results in Fig. 2A and Table 2 confirm previous observations [4,6,10,11] of almost no detectable viral cDNA integration in LEDGF/p75-knockdown cells (HeLaP4/shp75Cl15 cells [27]) infected by a WT HIV-1 (in this case at a multiplicity of infection (MOI) of 1.0). On the other hand, when the LEDGF/p75-knockdown cells were infected by a ΔRev HIV-1 at the same MOI, an average of about 4 integration events were observed per cell (Fig. 2A and Table 2, and see also Levin et al. [14]). These integration levels were greatly stimulated by the addition of increasing amounts of the INS peptide (Fig. 2A and Table 2). Such stimulation of integration was observed in LEDGF/p75-knockdown cells as well as in WT HeLa P4 cells infected with the WT or ΔRev viruses (Fig. 2A and Table 2). As many as 11.0 integration events in average per cell were observed when LEDGF/p75-knockdown cells were infected with WT virus at a MOI of 1.0 in the presence of 200 μM INS. However, when these cells were infected under the same experimental conditions with the ΔRev virus, the integration reached a high value of an average of 17.0 integration events per cell (Fig. 2A and Table 2).

**Table 2:** Summary of the results described in Figure 2.

		INS [μM]							
**Cells**	**Virus (MOI 1)**	**0**	**1**	**2.5**	**12.5**	**62.5**	**100**	**150**	**200**

HeLa P4	WT	1.02 ± 0.08	1.04 ± 0.07	1.25 ± 0.09	2.16 ± 0.11	3.30 ± 0.14	5.16 ± 0.20	9.70 ± 0.43	18.93 ± 0.74
	
	ΔRev	8.82 ± 0.37	9.13 ± 0.34	9.65 ± 0.41	10.98 ± 0.43	13.83 ± 0.61	16.66 ± 0.71	21.99 ± 0.94	30.16 ± 1.12

LEDGF/p75-knockdown	WT	0.04 ± 0.01	0.08 ± 0.01	0.17 ± 0.02	0.39 ± 0.03	0.69 ± 0.05	1.23 ± 0.10	3.32 ± 0.14	11.60 ± 0.57
	ΔRev	3.93 ± 0.17	4.05 ± 0.21	4.44 ± 0.27	5.21 ± 0.38	6.92 ± 0.47	8.30 ± 0.63	10.56 ± 0.74	16.80 ± 1.01

Values represent an average number of integrations per cell.

The results in Fig. 2A, show, as was reported previously [14,17,25], that infection of HeLa P4 cells by the WT virus (in the absence of INS) results in only 1.0 integration event (in average) per cell. This value increased to as high as 19.0 integration events (in average) per cell in the presence of 200 μM INS and to 30.0 integration events (in average) per cell following infection of the INS-treated HeLa P4 cells with ΔRev virus. The degree of viral cDNA integration was directly proportional to the concentrations of the INS added (Fig. 2A). Quantitative analysis of the total amount of viral cDNA in cells infected with WT or ΔRev HIV-1, both at a MOI of 1.0, revealed the presence of about 30.0 to 35.0 copies (in average) per cell (Fig. 2B). It appears therefore that a value of 30.0 integration events (in average) per cell--in the case of HeLa P4 cells treated with 200 μM INS and infected by a ΔRev HIV-1--reflects integration of practically all of the available viral cDNA copies. The number of cDNA copies generated (in average) per infected cell are not linearly correlated to the MOI added as was revealed by estimating the amount of viral cDNA copies per cell in cells infected by increasing MOIs (see Additional file 1 and Additional file 2, Fig. S1).

**Figure 3:**
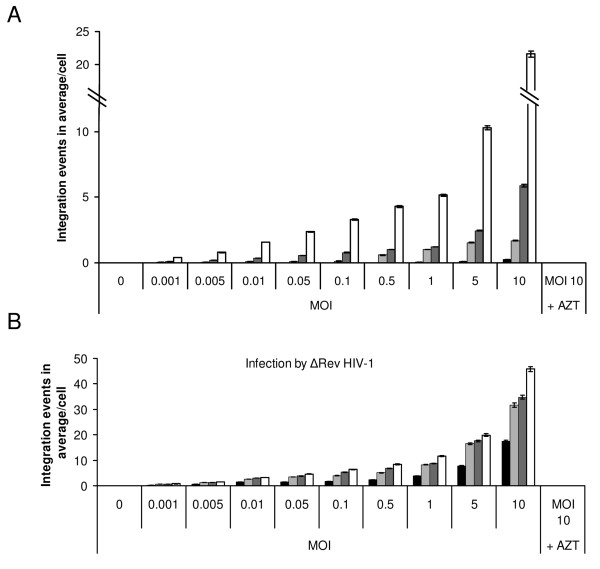
**Effect of increasing HIV-1 MOIs on integration levels in infected wt and LEDGF/p75-knockdown HeLa P4 cells**. HeLa P4 and HeLaP4/shp75Cl15 (LEDGF/p75-knockdown) cells were incubated with or without 100 μM INS and infected at the indicated MOIs with **(A) **WT or **(B) **ΔRev HIV-1. The average number of integration events per cell was estimated as described in Methods. Black shading and dark grey shading are infected LEDGF/p75-knockdown cells without or with INS treatment, respectively; light grey shading and white shading are infected HeLa P4 cells without or with INS treatment, respectively. AZT was used at 2 μM concentration.

In the absence of INS, practically no integration of viral cDNA was observed in the LEDGF/p75-knockdown cells, even when infected at high MOI (10.0) by the WT HIV-1 (Fig. 3A and Table 3). On the other hand, an increase in the degree of integration was observed the when LEDGF/p75-knockdown cells were infected with increasing amounts of WT HIV-1, reaching about 7.0 integration events in average per cell at a MOI of 10.0, in the presence of 100 μM INS (Fig. 3A and Table 3). The same increase was observed, but to a much higher degree of integration, when WT HeLa P4 cells were infected with increasing amounts of WT HIV-1 in the presence of INS (Fig. 3A and Table 3). A clear correlation between the amount of HIV-1 added and the degree of integration was observed when the same experiments were performed using the ΔRev virus (Fig. 3B and Table 3). As many as 50.0 and 23.0 integration events in average per cell were obtained following infection of WT HeLa P4 and LEDGF/p75-knockdown cells respectively by the ΔRev HIV-1 at a MOI of 10.0, in the presence of 100 μM INS (Fig. 3B and Table 3).

**Table 3:** Summary of the results described in Figure 3.

		MOI									
		
Cells and peptides	Virus	0	0.001	0.005	0.01	0.05	0.1	0.5	1	5	10
HeLa P4	WT	0	0.05 ± 0.01	0.06 ± 0.01	0.09 ± 0.01	0.10 ± 0.01	0.12 ± 0.01	0.51 ± 0.02	1.02 ± 0.08	1.53 ± 0.09	1.68 ± 0.11
	
	ΔRev	0	0.67 ± 0.04	1.34 ± 0.10	3.22 ± 0.15	3.42 ± 0.16	3.95 ± 0.18	5.13 ± 0.23	8.79 ± 0.37	17.59 ± 0.89	52.76 ± 2.21

LEDGF/p75-knockdown	WT	0	0	0.01 ± 0.00	0	0	0	0.01 ± 0.00	0.04 ± 0.01	0.08 ± 0.01	0.23 ± 0.03
	
	ΔRev	0	0.30 ± 0.03	0.60 ± 0.04	1.43 ± 0.08	1.52 ± 0.07	1.76 ± 0.09	2.28 ± 0.10	3.91 ± 0.13	7.83 ± 0.47	23.48 ± 1.34

HeLa P4 + INS 100 μM	WT	0	0.39 ± 0.02	0.78 ± 0.02	1.57 ± 0.09	2.35 ± 0.14	3.30 ± 0.20	4.29 ± 0.35	5.14 ± 0.44	10.29 ± 0.99	30.86 ± 2.14
	
	ΔRev	0	0.76 ± 0.03	1.52 ± 0.08	3.04 ± 0.14	4.56 ± 0.38	6.39 ± 0.71	8.30 ± 0.76	11.63 ± 1.01	19.93 ± 1.64	52.37 ± 3.42

LEDGF/p75-knockdown + INS 100 μM	WT	0	0.09 ± 0.01	0.19 ± 0.02	0.37 ± 0.02	0.56 ± 0.03	0.78 ± 0.06	1.02 ± 0.09	1.22 ± 0.10	2.44 ± 0.18	7.33 ± 0.42
	
	ΔRev	0	0.63 ± 0.04	1.26 ± 0.11	2.52 ± 0.18	3.79 ± 0.26	5.30 ± 0.44	6.89 ± 0.61	8.27 ± 0.74	16.54 ± 1.33	49.61 ± 3.11

Values represent an average number of integrations per cell.

Similar to INS, the INrs are IN-derived peptides which promote dissociation of the Rev-IN complex [17,22,24]. Therefore, in light of the above results, it was of interest to find out whether the INrs would also stimulate integration of viral cDNA in LEDGF/p75-knockdown cells. In contrast to the INS, the INrs do not interact with IN and therefore do not affect its enzymatic activity [22].

**Figure 4:**
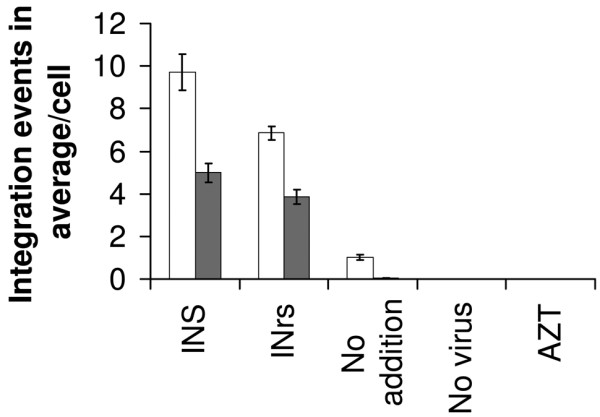
**INr peptides stimulate viral cDNA integration in LEDGF/p75-knockdown cells**. HeLa P4 (□) and HeLaP4/shp75Cl15 (LEDGF/p75-knockdown) (■) cells were incubated with or without 100 μM INS or INr and infected with wt HIV-1 at a MOI of 1.0. AZT was used at 2 μM concentration. The average number of integration events per cell was estimated as described in Methods.

From the results presented in Fig. 4 it is clear that the INrs were also able to significantly stimulate integration in LEDGF/p75-knockdown cells, most probably due to their ability to promote dissociation of the intracellular Rev-IN complex [17,22,24]. The extent of INr stimulation of integration levels was lower than that obtained by the INS peptide, probably due to their inability to enhance the enzymatic activity of the IN itself [22].

### Productive virus infection is greatly stimulated by the INS and INr peptides in LEDGF/p75-knockdown cells

**Figure 5:**
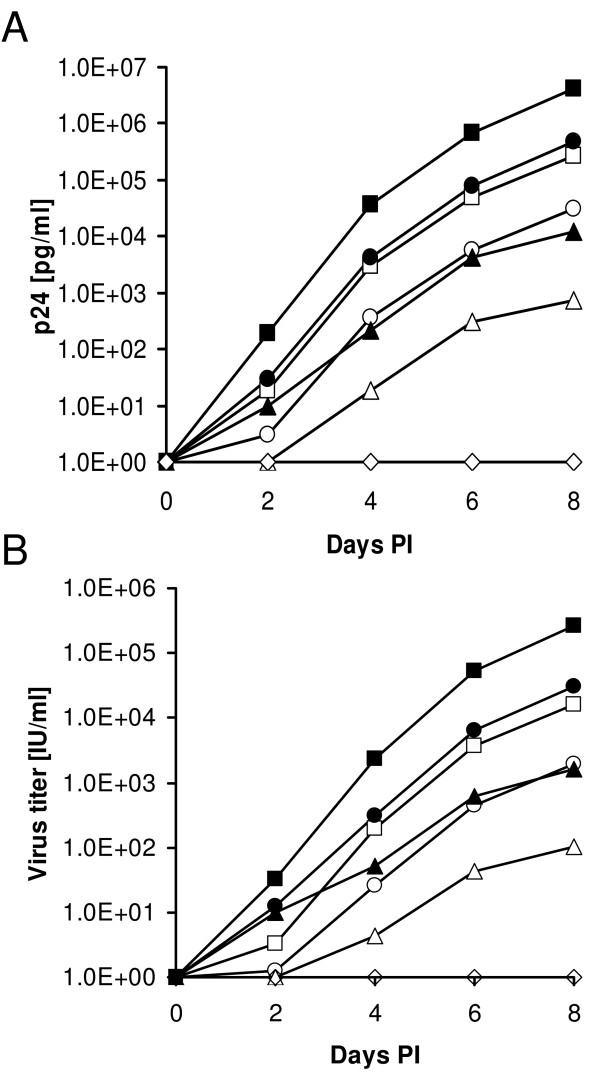
**Stimulation of p24 and virus production in LEDGF/p75-knockdown cells by the INS and INr peptides**. Sup-T1 and Sup-T1/TL3 (LEDGF/p75-knockdown) cells were incubated with or without 100 μM INS or INr and then infected with wt HIV-1 at a MOI of 0.01. The amount of viral p24 **(A) **and of infectious virus **(B) **was estimated every 2 days post-infection (PI) as described in Methods. ■, ● and ▲ represent Sup-T1 cells treated with INS, INr or not treated, respectively; □, ○ and Δ represent LEDGF/p75-knockdown cells treated with INS, INr or not treated, respectively; ◊ are non-infected cells.

The INS and INr peptides were also able to support high productive virus infection in LEDGF/p75-knockdown cells (Fig. 5), probably due to their ability to promote an increase in viral cDNA integration events in these cells. Production of both p24 (Fig. 5A) and infectious viruses (Fig. 5B) reached, in LEDGF/p75-knockdown cells and in the presence of the INr peptides, the same level as in infected, non-treated, WT HeLa P4 cells (Fig 5). Furthermore, even higher levels of p24 and virus production were obtained following addition of INS to the virus-infected LEDGF/p75-knockdown and wt HeLa P4 cells (Fig. 5A and 5B).

## Discussion

The results of the present work demonstrate that HIV-1 is able to efficiently infect cells which lack the cellular LEDGF/p75 protein, the presence of which is considered to be essential for productive infection [4,6,10,11]. However, infection of LEDGF/p75 knockdown cells occurs only in the presence of INS [25] or INr [17,22,24] peptides which promote dissociation of the Rev-IN complex, formed in the infected cells [14,17,18,22,24]. Following Rev-IN dissociation viral cDNA integration as well as virus production can reach, in LEDGF/p75-knockdown cells, even higher levels than those obtained in WT cells. The fact that viral cDNA integration can occur in LEDGF/p75-knockdown cells provided that the cells are infected by the ΔRev virus has already been demonstrated [14]. These results further supports the view that integration, and consequently infection, in LEDGF/p75-knockdown cells, is blocked by the inhibitory Rev. Infection by the ΔRev HIV-1 does not lead to productive infection due to the absence of Rev whose presence is required for nuclear export of unspliced and partially spliced viral RNA molecules [15].

**Figure 6:**
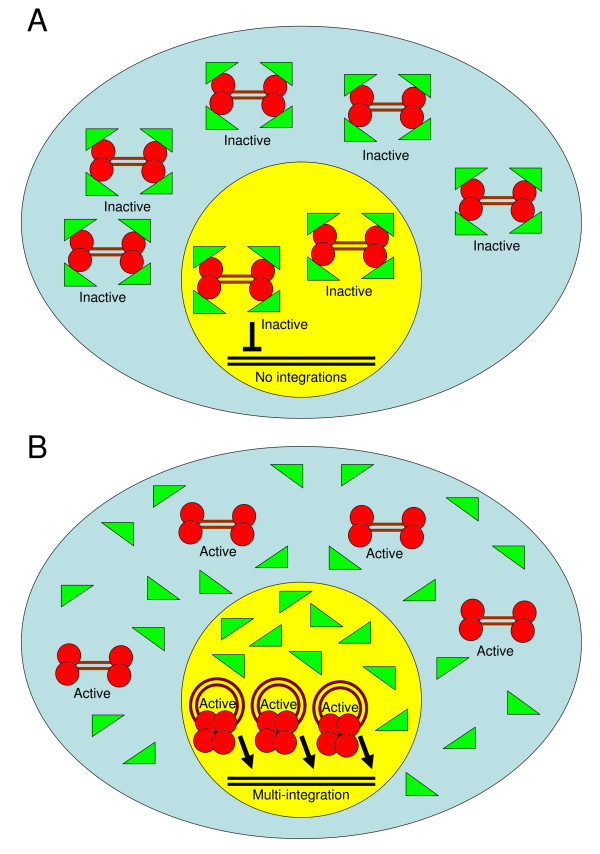
**Schematic model of the process of viral cDNA integration in wt and LEDGF/p75-knockdown cells. (A) **In LEDGF/p75-knockdown cells, an early expressed Rev (green triangle), from unintegrated viral DNA (brown double line), binds and inactivate all viral IN molecules (red circle) (in both the cytoplasm (light blue) and nucleus (yellow)) before integration occurs (due to the absence of LEDGF/p75 which supports rapid integration). **(B) **Addition of INS or INr peptides promotes dissociation of the inhibitory Rev-IN complexes, allowing the IN, which bound to viral DNA, to mediate integration into the host genome (black double line).

The way by which the interplay between the LEDGF/p75 and Rev proteins regulates integration of the viral cDNA has been described previously [14]. Infection by HIV-1 results in most cases in the integration of an average of 1 to 2 cDNA molecules per cell [14,17,22,25,28]. This is despite the fact that a large number (between 20 and 30 molecules) of cDNA remain unintegrated [28,29]. Our previous results [14,17] indicated that this is due to the fact that the large majority of the viral IN molecules, which catalyze the integration reaction, are inactive as a result of their interaction with the Rev protein [14,17,18,22]. It is possible, however, that the few integration events that do occur in virus-infected cells are mediated by IN molecules which were translocated, as IN-DNA complexes, into the nucleus before sufficient early Rev was expressed and thus escaped its inhibitory effect [14,18]. These active IN molecules then interact with the nucleus-localized LEDGF/p75 protein, which paves the way for the IN-DNA complexes to the host chromosomal DNA [1,5,13,30]. From our present results it appears that the resistance of LEDGF/p75-knockdown cells to HIV-1 infection and particularly the absence of any cDNA integration events in such cells is due to the inhibitory effect of Rev [4,6,10,11]. Due to the absence of the LEDGF/p75 protein in these cells, all of the IN molecules are available for interaction with Rev, resulting in the formation of inactive Rev-IN complex and complete inhibition of cDNA integaration (Fig. 6A and Levin et al. [17,18,22]). Promotion of the Rev-IN complex dissociation by the INr or INS peptides results in reactivation of the IN enzymatic activity, thus allowing relatively efficient integration and virus production in LEDGF/p75-knockdown cells (Fig. 6B).

According to this view, the Rev protein plays a major role in restricting, in WT cells, and totally inhibiting, in LEDGF/p75-knockdown cells, the integration of viral cDNA and consequently virus replication and production. In addition to regulation by Rev, integration is probably regulated by the enzymatic activity of IN itself since stimulation of this activity by INS resulted in further stimulation of integration [25].

## Methods

### Protein expression and purification

Expression and purification of the histidine-tagged IN and LEDGF/p75 expression vectors, were a generous gift from Prof. Engelman, Dana-Farber Cancer Institute and Division of AIDS, Harvard Medical School, Boston, MA, USA), were performed essentially as described previously [31,32].

### Peptide synthesis and purification

Peptides were synthesized on an Applied Biosystems (ABI; Carlsbad, California, USA) 433A peptide synthesizer and purification was performed on a Gilson HPLC using a reverse-phase C8 semi-preparative column (ACE, Advanced Chromatography Technologies, Aberdeen AB25 1DL, United Kingdom) as described in Levin et al. [22].

### ELISA-based binding assays

Protein-peptide, protein-protein and protein-DNA binding was estimated using an ELISA-based binding assay exactly as described previously [33]. Briefly, Maxisorp plates (Nunc) were incubated at room temperature for 2 h with 200 ml of 10 μg/ml synthetic peptide/recombinant proteins in carbonate buffer. After incubation, the solution was removed, the plates were washed three times with PBS, and 200 μl of 10% BSA (Sigma) in PBS (w/v) was added and the plates were further incubated for 2 h at room temperature. After rewashing with PBS, the tested BSA-biotinilated (Bb) peptide or protein (alone or biotinilated) was added for a further 1-h incubation at room temperature. Following three washes with PBS, the concentration of bound molecules was estimated following the addition of streptavidin-horseradish peroxidase (HRP) conjugate (Sigma), as described previously [34]. The enzymatic activity of HRP was estimated by monitoring the product's optical density (OD) at 490 nm using an ELISA plate reader (Tecan Sunrise, Männedorf, Switzerland). Each measurement was performed in duplicate. Estimation of complex dissociation was performed as follow: after binding of the first protein to the maxisorp plate, the binding partner was incubated for 1 h at room temperature and after three washes in PBS, the dissociating component was added and its binding to the complex, as well as the remaining bound complex, were estimated separately as described above.

### Cells

Monolayer adherent HEK293T, and HeLa MAGI cells (TZM-bl) [35,36], as well as HEK293T cells over-expressing Rev (Rev10^+ ^cells [37]), were grown in Dulbecco's Modified Eagle's Medium (DMEM). The T-lymphocyte cell lines Sup-T1 and Sup-T1/TL3 were grown in RPMI 1640 medium. Cells other than the Rev10^+^, HeLaP4/shp75Cl15 and Sup-T1/TL3 cells were provided by the NIH Reagent Program, Division of AIDS, NIAID, NIH (Bethesda, MD, USA). The various cells were incubated at 37°C in a 5% CO_2 _atmosphere. All media were supplemented with 10% (v/v) fetal calf serum, 0.3 g/l L-glutamine, 100 U/ml penicillin and 100 U/ml streptomycin (Biological Industries, Beit Haemek, Israel). HeLaP4/shp75Cl15 cells (a generous gift from Prof. Debyser, Molecular Medicine, K.U. Leuven, Flanders, Belgium), were grown as described in Vandekerckhove et al. [27]. Sup-T1/TL3 cells, (a generous gift from Prof. Poeschla Department of Molecular Medicine, Mayo Foundation, Rochester, MN, USA), were grown as described in Llano et al. [5].

### Viruses

The wt HIV-1 (HXB2 [38]) was generated by transfection into HEK293T cells [39]. The ΔRev pLAIY47H2 [40] HIV was generated by transfection into Rev10^+ ^cells [37]. Viruses were harvested and stored as described previously [22]. The pLAIY47H2 [40] viruses were a generous gift from Prof. Berkhout (Department of Human Retrovirology, Academic Medical Center, University of Amsterdam, The Netherlands). Virus stocks were concentrated by ultracentrifugation (25,000rpm at 15°388 for 105 min) using Beckman SW28 rotor [41]. All viral stocks were treated with 50 U/ml DNase for 1 h at 37 °C in order to eliminate excess of viral DNA plasmid

### Infection of cultured lymphocyte cells with HIV-1

Cultured lymphocytes (1 × 10^5^) were centrifuged for 5 min at 500 g and after removal of the supernatant, the cells were resuspended in 0.2 to 0.5 ml RPMI 1640 medium containing virus at different MOIs. Following absorption for 2 h at 37°C, the cells were washed to remove unbound virus and then incubated at the same temperature for an additional 2 days [23].

### Study of *in-vivo *protein-protein interactions using the Co-IP methodology

The Co-IP experiments were conducted essentially as described previously [42] with the following modifications. Briefly, cells were infected at a MOI of 15 of the indicated viruses. Infected cells were harvested at different times post-infection, washed three times with PBS and lysed by the addition of PBS containing 1% (v/v) Triton X-100 for whole-cell lysate. Half of the lysate was subjected to SDS-PAGE (an E-PAGE™ *48 8*% High-Throughput Pre-Cast Gel System (Invitrogen)) and immunoblotted with either monoclonal anti-Rev antibody (α-Rev) [43], antiserum raised against IN amino acids 276-288 (α-IN) (NIH AIDS Research & Reference Reagent Program catalog number 758), anti-LEDGF/p75 (α-LEDGF/p75) (R&D Systems, Minneapolis, MN, USA) or anti-actin (α-Actin) antibody (Santa Cruz), and complementary HRP-conjugated antibodies (Jackson ImmunoResearch, West Grove, PA, USA) as second antibodies.

The remaining lysate was incubated for 1 h at 4°C with either the α-Rev, α-IN, α-LEDGF/p75 or α-Actin antibodies. Following 3-h incubation at 4°C with protein G-agarose beads (Santa Cruz Biotechnology, Santa Cruz, CA, USA), the samples were washed three times with PBS containing 1% (v/v) Nonidet P-40. SDS buffer was added to the samples and after boiling and SDS-PAGE (an E-PAGE™ *48 8*% High-Throughput Pre-Cast Gel System (Invitrogen)), the membranes were immunoblotted with either α-Rev, α-IN, α-LEDGF/p75 or α-Actin antibodies, and complementary HRP-conjugated antibodies (Jackson) as second antibodies. When peptides were used, cells were incubated with 150 μM of the indicated peptide for 2 h prior to infection.

### Quantitative determination of the average copy numbers of HIV-1 DNA integrated into the cellular genome

The integration reaction was estimated essentially as described previously [23]. Briefly, following incubation of the indicated peptides with Sup-T1 cells for 2 h, the cells were infected at the indicated MOI. Integrated HIV-1 sequences were amplified by two PCR replication steps using the HIV-1 LTR-specific primer (LTR-Tag-F 5'-ATGCCACGTAAGCGAAACTCTGGCTAACTAGGGAACCCACTG-3') and Alu-targeting primers (first-Alu-F 5'-AGCCTCCCGAGTAGCTGGGA-3' and first-Alu-R 5'-TTACAGGCATGAGCCACCG-3') [44]. Alu-LTR fragments were amplified from 10 ng of total cell DNA in a 25-μl reaction mixture containing 1× PCR buffer, 3.5 mM MgCl_2_, 200 μM dNTPs, 300 nM primers, and 0.025 U/μl of Taq polymerase. The first-round PCR cycle conditions were as follows: a DNA denaturation and polymerase activation step of 10 min at 95°C and then 12 cycles of amplification (95°C for 15 s, 60°C for 30 s, 72°C for 5 min).

During the second-round PCR, the first-round PCR product could be specifically amplified using the Tag-specific primer (Tag-F 5'-ATGCCACGTAAGCGAAACTC-3') and the LTR primer (LTR-R 5'-AGGCAAGCTTTATTGAGGCTTAAG-3') designed by PrimerExpress (ABI) using the default settings. The second-round PCR was performed on 1/25th of the first-round PCR product in a mixture containing 300 nM of each primer and 12.5 μl 2× SYBR Green Master Mix (ABI) at a final volume of 25 μl, and run on an ABI PRIZM 7700. The second-round PCR cycles began with DNA denaturation and a polymerase-activation step (95°C for 10 min), followed by 40 cycles of amplification (95°C for 15 s, 60°C for 60 s).

To generate a standard calibration curve, the SVC21 plasmid containing the full-length HIV-1_HXB2 _viral DNA was used as a template. In the first-round PCR, the LTR-Tag-F and LTR-R primers were used and the second-round PCR was performed using the Tag-F and LTR-R primers. The standard linear curve was in the range of 5 ng to 0.25 fg (*R *= 0.99). DNA samples were assayed with quadruplets of each sample (Additional file 3, Fig. S2). For further experimental details, see Rosenbluh et al. [23] see also [45]. The cell equivalents in the sample DNA were calculated based on amplification of the 18 S gene by real-time PCR as described in Field et al. [46].

### Quantitative determination of total viral DNA copies

Total viral DNA was estimated using SYBR Green real-time quantitative PCR at 12 h post-infection from the total extract of infected cells. DNA was isolated by the phenol-chloroform method. Briefly, DNA samples (1 μg) were added to 95 μl containing 1× Hot-Rescue Real Time PCR Kit-SG (Diatheva s.r.l, Fano, Italy), and 100 nM of each primer-binding-site primer: F5 (5' primer, 5'-TAGCAGTGGCGCCCGA-3') and R5 (3' primer, 5'-TCTCTCTCCTTCTAGCCTCCGC -3'). All amplification reactions were carried out in an ABI Prism 7700 Sequence Detection System: one cycle at 95°C for 10 min, followed by 45 cycles of 15 s at 95°C and 35 s at 68°C. In each PCR run, three replicates were performed. All other details are exactly as described in Casabianca *et al*. [47].

### HIV-1 titration by multinuclear activation of a galactosidase indicator (MAGI) assay

Quantitative titration of HIV-1 was carried out using the MAGI assay, as described previously [36]. Briefly, TZM-b1 cells were grown in 96-well plates at 10^4 ^cell/well and incubated for 12 h at 37°C. Peptides were then added and after an additional 2 h of incubation, the cells were infected with 50 μl of serially diluted HIV-1. Cultured cells were fixed 2 days post-infection and β-galactosidase was estimated [23,48,49]. Blue cells were counted under a light microscope at 200× magnification. It should be noted using this assay system may results in slightly higher titer of virus due to leakiness.

### Quantitative estimation of HIV-1 infection by determination of extracellular p24

The amount of p24 protein was estimated in the cell medium exactly as described previously [23].

All experiments were repeated three to four times and the differences between the obtained results never exceeded ± 10%.

## Authors' contributions

AL designed and performed the experiments, analyzed data and contributed to writing the paper; ZH performed peptide synthesis and purification; AF designed the study, and contributed to the writing; AL designed the study, contributed to the writing of the paper and coordinated the study. All authors have read and approved the manuscript.

## Competing interests

The authors declare that they have no competing interests.

## Supplementary Material

Additional file 1 **A non linear correlation exist between the HIV-1 MOIs and the amount of cDNA copies calculated per virus per infected cell**. Additional data demonstrating the correlation between the MOI used and the amount of cDNA copies that can produce be the virus per infected cell. Click here for file

Additional file 2 **The correlation between the amounts of infected virus added and the cDNA copies in infected cells**. **(A) **HeLa P4 cells (1 × 10^5^) were incubated by the wt HIV-1 at the indicated MOIs. The average amount of viral cDNA copies per cell was estimated as described in Methods. **(B) **The correlation between the calculated average amount of cDNA copies per virus per cell and the MOIs used for infection. The average numbers of cDNA copies per virus per cell were estimated based on the results depicted in **(A) **divided the MOI namely, the average number of virions used to infect each cell. Click here for file

Additional file 3 **Calibration of the quantitative Real time measurement of integration events**. **(A) **Dissociation curve of the integration sample from infected cells (in red) vs. a sample from the standard used for this real time PCR assay (green). **(B) **Standard curve used for the estimation of the average number of integration events. Click here for file
