# Supplementary material for: Peptides derived from the HIV-1 integrase promote HIV-1 infection and multi-integration of viral cDNA in LEDGF/p75-knockdown cells
Source: Virol J. 2010 Aug 2;7:177. doi: 10.1186/1743-422X-7-177 (PMC2924314; doi:10.1186/1743-422X-7-177)
Supplement: Additional file 1 — A non linear correlation exist between the HIV-1 MOIs and the amount of cDNA copies calculated per virus per infected cell. Additional data demonstrating the correlation between the MOI used and the amount of cDNA copies that can produce be the virus per infected cell. [file 1743-422X-7-177-S1.DOC]

**A non linear correlation exist between the HIV-1 MOIs and the amount of cDNA copies calculated per virus per infected cell**

The results in Fig. 3 (see also Table 3) show that on the average about 6 integration events per cell were obtained following infection by MOI of 0.1 of INS treated cells. Under these conditions only about 10% of the cell should be infected thus indicating about 60 integrations per infected cells. This may appear surprising especially in the light that infection by MOI of 1.0—which should result infection of most of the cells in the population—resulted in only 11.5 integration per cell (Fig. 3). Certainly this raise the question how there are sufficient cDNA copies to allow 60 integration events per cell following infection by MOI of 0.1. This can be explained by the results depicted in Fig. S1A which show that a non-linear correlation exists between the MOIs added and the average amount of viral cDNA copies observed per cell (Fig. S1A). Interestingly, a calculation of the average amount of viral cDNA copies per virus per infected cell (Fig S1B) revealed that the higher the MOIs, the less copies of cDNA per virus are generated in each infected cell (Fig. S1B). It is clear that following infection by 10 MOI only about 7.0 cDNA copies in average were calculated to be generated from each virus per infected cell, where following infection by MOI of 0.1 as many as 53 copies (in average) of cDNA can be generated per virus per infected cells (Fig. S1B). This phenomenon may result either from an interference of virus infection at relatively high titer especially due to resistance to superinfection (see ref [1, 2]) or interference with the reverse transcription mechanism related to the efficiency of the RNase H activity whose activity should be dependent on the enzyme concentration [3, 4]. Further studies are required to elucidate the exact mechanism responsible to this phenomenon.

References

1. Levin A, Hayouka Z, Friedler A, Brack-Werner R, Volsky DJ, Loyter A: **A novel role for the viral Rev protein in promoting resistance to Super-infection by Human Immunodeficiency Virus type 1.** *J Gen Virol* 2010, **91:**1503-1513.

2. Sloan RD, Donahue DA, Kuhl BD, Bar-Magen T, Wainberg MA: **Expression of Nef from unintegrated HIV-1 DNA downregulates cell surface CXCR4 and CCR5 on T-lymphocytes.** *Retrovirology* 2010, **7:**44.

3. DeStefano JJ, Buiser RG, Mallaber LM, Myers TW, Bambara RA, Fay PJ: **Polymerization and RNase H activities of the reverse transcriptases from avian myeloblastosis, human immunodeficiency, and Moloney murine leukemia viruses are functionally uncoupled.** *J Biol Chem* 1991, **266:**7423-7431.

4. Schultz SJ, Champoux JJ: **RNase H activity: structure, specificity, and function in reverse transcription.** *Virus Res* 2008, **134:**86-103.
