# Supplementary figures and images for: Peptides derived from the HIV-1 integrase promote HIV-1 infection and multi-integration of viral cDNA in LEDGF/p75-knockdown cells
Source: Virol J. 2010 Aug 2;7:177. doi: 10.1186/1743-422X-7-177 (PMC2924314; doi:10.1186/1743-422X-7-177)

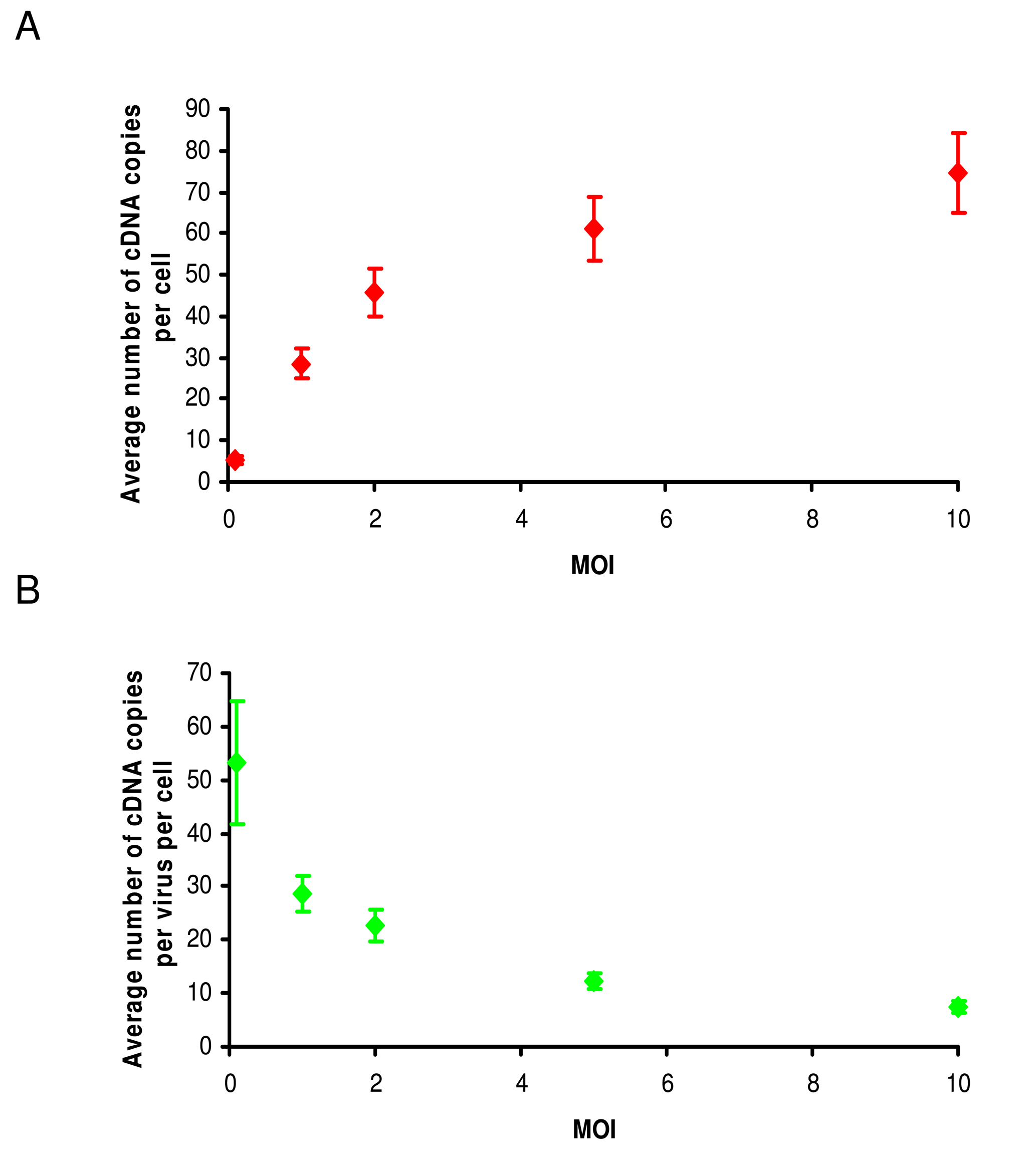

Supplement: Additional file 2 — The correlation between the amounts of infected virus added and the cDNA copies in infected cells. (A) HeLa P4 cells (1 × 10^5^) were incubated by the wt HIV-1 at the indicated MOIs. The average amount of viral cDNA copies per cell was estimated as described in Methods. (B) The correlation between the calculated average amount of cDNA copies per virus per cell and the MOIs used for infection. The average numbers of cDNA copies per virus per cell were estimated based on the results depicted in (A) divided the MOI namely, the average number of virions used to infect each cell. [file 1743-422X-7-177-S2.TIFF]

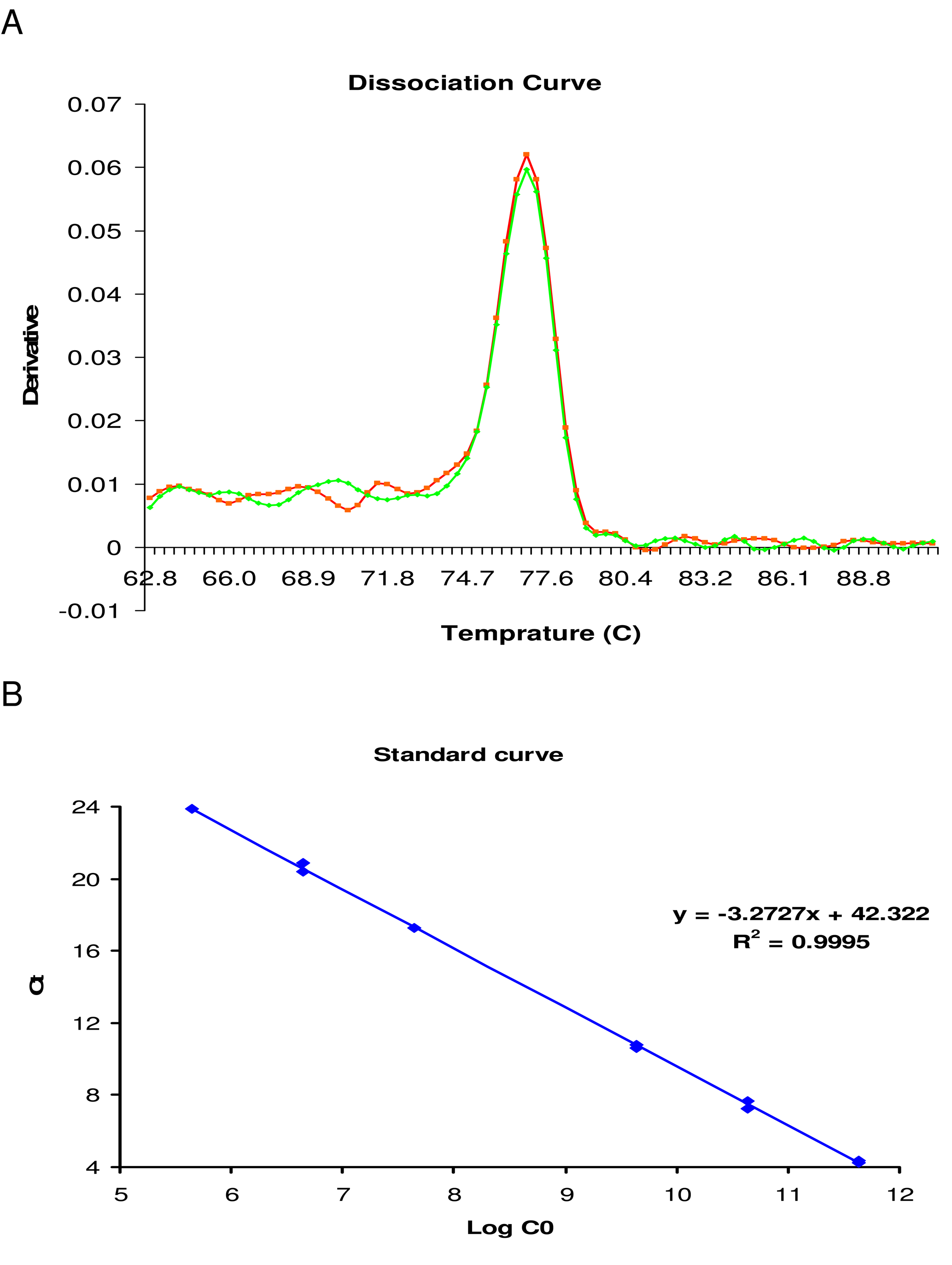

Supplement: Additional file 3 — Calibration of the quantitative Real time measurement of integration events. (A) Dissociation curve of the integration sample from infected cells (in red) vs. a sample from the standard used for this real time PCR assay (green). (B) Standard curve used for the estimation of the average number of integration events. [file 1743-422X-7-177-S3.TIFF]
